# Spotting Signs of Autism in 3-Year-Olds: Comparing Information from Parents and Preschool Staff

**DOI:** 10.1007/s10803-018-3821-5

**Published:** 2018-11-21

**Authors:** Elisabeth Nilsson Jobs, Sven Bölte, Terje Falck-Ytter

**Affiliations:** 1 Uppsala Child and Baby Lab, Department of Psychology, Uppsala University, 751 42 Uppsala, Sweden; 2 Center of Neurodevelopmental Disorders (KIND), Division of Neuropsychiatry, Department of Women’s & Children’s Health, Karolinska Institutet, Stockholm, Sweden; 3 Center of Psychiatry Research, Stockholm County Council, Stockholm, Sweden; 4 Child and Adolescent Psychiatry, Stockholm County Council, Stockholm, Sweden; 5 Swedish Collegium for Advanced Study (SCAS), Thunbergsvägen 2, SE-752 38 Uppsala, Sweden

**Keywords:** Autism spectrum disorder, CBCL, C-TRF, Preschool, Child development

## Abstract

Preschool informants may provide valuable information about symptoms of autism spectrum disorder (ASD) in young children. We compared the diagnostic accuracy of ratings by preschool staff with those by parents of 3-year-old children using the Achenbach System of Empirically Based Assessment Preschool Forms. The sample consisted of 32 children at familial risk for ASD without diagnosis, 10 children at risk for ASD with diagnosis, and 14 low-risk typically developing controls. Preschool staff ratings were more accurate than parent ratings at differentiating children with and without ASD, and more closely associated with clinician-rated symptoms. These results point to the value of information from preschool informants in early detection and diagnostic assessments.

Symptoms of autism spectrum disorder (ASD) usually emerge during the second year of life (Lord et al. 2012a; Macari et al. 2018). It is recommended that the assessment of ASD is supported by structured clinical observations with the Autism Observation Schedule-2 (ADOS-2) and by the Autism Diagnostic Interview-Revised (ADI-R; Lord et al. 2012b; Rutter et al. 2003, 2008; Huerta and Lord 2012; Falkmer et al. 2013). In addition, information from other informants such as preschool staff about child behavior in preschool is potentially valuable (Dereu et al. 2012; Ivanova et al. 2011; Gustafsson et al. 2017). It has been suggested that teachers can evaluate individual children more accurately than parents because they can compare the individual child with a range of peers (Reed and Osborne 2013). Research has shown that although both parents and preschool staff are accurate at differentiating children with ASD from community samples and children with typical development (Larsen et al. 2018; Schanding et al. 2012; Stickley et al. 2017), preschool staff are better at discriminating children with ASD and children with other developmental concerns (Aldridge et al. 2012; Stickley et al. 2017). These findings were all based on the Social Responsiveness Scale (SRS; Constantino 2002; Constantino and Gruber 2012). Other work using this scale indicates that compared to parents, teachers’ ratings differentiate children more accurately in terms of ADOS-classification and show higher correlations with independently assessed symptom severity scores on the ADOS (Lord et al. 1999, 2012b; Gotham et al. 2007; Duvekot et al. 2015; Reszka et al. 2014; Schanding et al. 2012). Available research in this area has mainly focused on children older than 3 years, and little is known about the accuracy of the information provided by preschool informants at younger ages.

To study early development in ASD, longitudinal studies of siblings are becoming increasingly common. Herein, younger children with an older sibling with autism are followed from an early age. About 14–20% of the high-risk children receive an ASD diagnosis, which can be compared to approximately 1–2% in the general population (CDC 2018; Messinger et al. 2013). Even in children of the high-risk group that do not fulfill criteria for ASD, both subclinical autism symptoms and other neurodevelopmental as well as behavioral problems are common (Charman et al. 2017; Kantzer et al. 2018; Ozonoff et al. 2014; Landa et al. 2013; Bussu et al. 2018; Messinger et al. 2013). Including siblings as a comparison group allows investigating whether informants can make distinctions between groups that both may have elevated symptom levels, and have a familial history of ASD. No previous study has used this approach to compare preschool and parent ratings of young children with risk of autism. However, Rescorla et al. (2017) evaluated parent information on autism symptoms at 24 months comparing siblings with ASD, siblings at risk but without ASD and a group of typically developing low risk controls. They found that parents could differentiate the autism group from the other groups.

In the current study, we looked closer at parents’ and preschool staff’s reporting on categorical and dimensional aspects of ASD in 3-year-olds, an age when autism symptoms are evident in most children affected by ASD (Zwaigenbaum et al. 2016). Rather than evaluating a questionnaire specifically focusing on autistic behaviors, we evaluated a broadband preschool assessment tool that also includes scales on autism related behaviors. We chose to focus on this measure as it gives clinicians comprehensive information about the child’s behavior at home and preschool, desirable in the assessment and differential diagnoses of neurodevelopmental disorders. Moreover, it is an efficient way to gain as much information at a low time cost for the preschool staff and caregivers as possible. One scale that is standardised for both parents and preschool staff is the Child Behavior Checklist 1.5–5 (CBCL 1.5–5) and the Caregiver-Teacher Report Form (C-TRF; Achenbach and Rescorla 2000, 2004), including scales on autism-related behavior as well as other developmental and behavioral concerns. We focused on the syndrome Withdrawn- and DSM-related Pervasive Developmental Problem (PDP) scales. The Withdrawn and the PDP scales on the parent CBCL 1.5–5 have been investigated in relation to ASD. Although clear group mean differences on these scales have been found in children with ASD, both compared to children with other developmental disorders and with typical development, the view upon the clinical utility of these scales varies (Rescorla et al. 2017; Muratori et al. 2011; Limberg et al. 2017; Havdahl et al. 2016; Myers et al. 2014; Narzisi et al. 2013). The C-TRF Withdrawn and the PDP scales have previously not been evaluated for preschool staff.

The purpose of this study was to compare parents’ and preschool staff’s ratings of autistic symptoms in young children in relation both to diagnostic assessments and to a gold-standard diagnostic instrument (the ADOS-2).

Specifically, the first aim of this study was to investigate if ratings on the CBCL 1.5–5 and C-TRF Withdrawn and PDP scales would discriminate between groups with and without ASD at 36 months. We hypothesized, based on previous findings on the SRS that both parents and preschool staff could differentiate between a low-risk group with typical development, a high-risk group without ASD and a group with ASD. However, we also expected that preschool staff ratings would be more accurate than parent ratings.

Our second aim was to investigate if variance of the ADOS-2 Comparison Scores (CS) could be predicted by scores on the CBCL 1.5–5 and C-TRF Withdrawn and PDP scales. Again, in line with findings on the SRS, we expected that results for both informants on these scales would predict variance in the ADOS-2 CS, but that preschool ratings would have a higher predictive value than parent ratings.

## Methods

### Participants

The project was approved by the Regional Ethical Board in Stockholm. Participants were part of the longitudinal Early Autism Sweden (EASE; smasyskon.se) sibling project, including siblings with high and low risk for ASD assessed at 36 months.

Out of the 91 children that were assessed at 36 months, 60 children had been rated both by parents and preschool staff. After exclusion of four participants (one control child having ASD; one child not fulfilling initial inclusion criteria, detected in retrospect; one participant having no diagnostic status at the time of analyses and; one participant being an statistical outlier), the final analysis was based on 56 participants.

The sample consisted of 32 (18 girls) participants with high-risk-for-ASD with no diagnosis (HR-noASD); 10 (5 girls) with high-risk-for-ASD with ASD diagnosis (HR-ASD); and 14 (7 girls) children with low risk for ASD (LR), i.e., typically developing controls. Seven children in the HR-noASD group had signs of ADHD-symptoms (observed by an experienced clinician throughout the day, both during formal assessment and during breaks in interaction with the parent) and one child of expressive and receptive language impairment defined as a T-score ≤ 35 on the Expressive and/or Receptive scale on the Mullen Scales of Early learning (MSEL; Mullen 1995). In the HR-ASD group one child had signs of ADHD, three children of either expressive or receptive language impairment and one of developmental delay (defined as Total composite IQ ≤ 70 on the MSEL). In the LR group there were no signs of other developmental concerns. Participant characteristics are presented in Table 1, which show that there was no group difference for age. For ADOS-2 CS, the HR-ASD group had higher scores than both HR-no and LR groups and reversely, the total scores on the Vineland ABC and the MSEL were lower in the HR-ASD group than in both other groups. There was no difference between the LR and HR-noASD groups on any measure.

**Table 1:** Sample characteristics

	LR n = 14			HR-noASD n = 32			HR-ASD n = 10			Group comparisons		
	M	SD	Range	M	SD	Range	M	SD	Range	F (df)	*p*	post hoc Bonferroni
Age, months	36.71	1.11	4.93	36.97	1.09	5.32	37.04	1.14	3.62	0.35 (2, 53)	.705	*ns*
ADOS-2 CS	2.64	1.34	4	3.22	1.74	7	7.1	1.66	5	25.90 (2, 53)	< .001	HR-ASD > (LR***; HR-noASD***)
MSEL tot	114.9	14.68	68	109.6	14	71	90.00	19.87	72	8.49 (2, 53)	.001	HR-ASD < (LR***; HR-noASD**)
VABScomp	98.07	10.96	40	93.75	7.57	31	82.30	12.25	44	8.59 (2, 53)	.001	HR-ASD < (LR***; HR-noASD**)

*LR* Low-risk siblings, *HR-noASD* High-risk sibling with no ASD, *HR-ASD* High-risk sibling with ASD, *ADOS-2* Autism Diagnostic Observation Schedule-2, *CS* Comparison Score, *MSEL tot* Mullen Scales of Early Learning total IQ, *VABScomp* Vineland Adaptive Behavior Scales, composite standard score

**p ≤ .01; ***p ≤ .001

### Procedure

Data collection was conducted between March 2014 and June 2017. All assessments took place during one day in a clinical setting. Diagnosis at 36 months was based on consensus of two experienced clinicians according to DSM-5 criteria (American Psychiatric Association 2013), based on information from the Autism Diagnostic Observation Schedule-2, module 1 or 2 (ADOS-2; Lord et al. 2012b); the Autism Diagnostic Interview-Revised (ADI-R; Rutter et al. 2003, 2008); the Vineland Adaptive Behavior Scales-2 (VABS-II; Sparrow et al. 2005; McDonald 2014; Mouga 2014) and the MSEL (Mullen 1995). The CBCL 1.5–5 and C-TRF ratings were collected independent of diagnostic decision-making.

The CBCL 1.5–5 was completed at home either just before or after the 36-month visit. Thirty-five CBCLs had been answered by the mother, 9 answered by the father and 12 by both parents. The C-TRF was distributed to preschool through the parents. The preschool informants consisted of 30 preschool teachers (with bachelor degree), 21 preschool care staff (with upper secondary level education), two with other background and in three cases information on informant profession was lacking. The preschool informants had known the child more than 6 months in 46 cases, less than 6 months in eight cases and familiarity was unknown in two cases. The ratings from preschool were sent directly to the research team by regular mail or they were returned via the parents at a scheduled visit in the EASE study. All parent and preschool ratings were completed before the diagnostic evaluation, why the informants were blind to the result of the diagnostic assessment.

### Measures

#### Cognitive Development

The MSEL (Mullen 1995) is a standardized measure of cognitive development from birth to 68 months of age. Scores are obtained from the subscales Fine motor-, Visual reception-, Expressive- and Receptive- language scales presented in T-scores and a total composite standard score as a proxy for IQ.

#### Adaptive Functioning

The VABS-II (Sparrow et al. 2005) is a semi-structured parent-report questionnaire covering four different domains: Communication, Daily Living Skills, Socialization and Motor Abilities. The scales are evaluated separately or as an overall ABC in standard scores (mean = 100, SD = 15), the latter applied in this study.

#### Autism Symptoms

The ADOS-2 (Lord et al. 2012b) is a semi-structured standardized assessment of communication, social interaction, play and restricted and repetitive behavior. The result is either presented as an algorithm score or as a CS, the latter enabling comparison between modules. CS were included in this study.

#### ASEBA Scales

The CBCL 1.5–5 and C-TRF (Achenbach and Rescorla 2000, 2004) consist of 100 questions that are rated 0, 1 or 2 and summed to scores on a Total problem scale, Internalizing and Externalizing scales, seven syndrome scales (Emotional reactivity, Anxious/depressed, Somatic complaints, Withdrawn, Sleep problems, Attention problems and aggressive behavior) or scales in line with DSM-criteria (Affective problems, Anxiety problems, Pervasive developmental problems, Attention deficit/Hyperactivity problems and Oppositional defiant problems). In this study, the syndrome scale Withdrawn and the DSM related Pervasive developmental problems (PDP) scale of the CBCL 1.5–5 and C-TRF (Achenbach and Rescorla 2000) were used. The PDP scale is identical to the DSM-ASD scale in the recent CBCL 1.5–5—C-TRF version (Achenbach 2014) apart from that the item “afraid of trying new things” that is not included in the DSM-ASD scale. The common and specific items of these subscales are presented in Table 2.

**Table 2:** Items within the CBCL 1.5–5/C-TRF subscales Withdrawn and PDP (Achenbach and Rescorla 2000)

CBCL—C-TRF Withdrawn	CBCL—C-TRF Pervasive developmental problems (PDP)
**Avoids looking others in the eye** **Doesn**’**t answer when people talk to him**/**her** **Seems unresponsive to affection** **Shows little affection toward people** **Withdrawn, doesn**’**t get involved with others** Acts young for age Refuses to play active games Shows little interest in things around him/her CBCL Constipated /C-TRF Apathetic^a^ CBCL Diarrhea/C-TRF Daydreams^a^	**Avoids looking others in the eye** **Doesn**’**t answer when people talk to him**/**her** **Seems unresponsive to affection** **Shows little affection toward people** **Withdrawn, doesn**’**t get involved with others** Doesn’t get along with other children Speech problem *Afraid to try new things* *Can*’*t stand things out of place* *Disturbed by any change in routine* *Rocks head or body* *Strange behavior* *Upset by new people or situations*

Bold = Same questions on both scales; Italics = Questions on RRBs, PDP scale

^a^Different questions depending on informant

### Analyses

Statistics were performed in SPSS 24 (IBMCorp 2016). Percentile confidence intervals 95%, based on 1000 bootstrap samples, were applied in all descriptive and main results. Prior to analysis, one participant was considered as an outlier based on extreme values for Mahalanobis distances (> 11), Cook’s distance (> 1) and standardized DFBeta (> 1) for both the Withdrawn and PDP subscales, and removed from the analyses. Due to the Withdrawn and PDP scales partly including the same items, regression analyses were conducted in pairs (i.e. CBCL 1.5–5 Withdrawn vs. C-TRF-Withdrawn; CBCL 1.5–5 PDP vs. C-TRF-PDP) for both linear as well as multi-nominal regression. In the linear regression we focused on the children at high risk for ASD (i.e., the HR-ASD and the HR-noASD groups together), excluding the LR group. We chose to focus on the HR group because it is homogenous in terms of familial history, recruitment type, and is expected to encompass a large variation in traits/symptoms. All analyses of the scales were based on raw scores. Results were reported by 2-tailed significance but given our directional hypothesis, trends were also reported.

#### Categorical Comparisons (Aim 1)

Comparison between groups on the Withdrawn- and PDP scales were conducted list-wise with one-way ANOVAs, one for each informant and subscale. Results were analysed with Brown Forsyth F*. Corrections of *p*-values for multiple comparisons were calculated by the method of False discovery rate (Benjamini and Hochberg 1995). Post hoc results were reported according to Games Howell’s test. For the prediction of group, prior to analyses, all predictor variables were tested for linearity of the logit (binary regression with the predictor variables analysed separately with the groups LR vs. HR-noASD; LR vs. HR-ASD; HR-noASD vs. HR-ASD as outcome variables). All interactions had greater values than .05 (*p*s > .22), indicating no violation of this assumption. Multi-nominal logistic regression was conducted with LR, HR-noASD and HR-ASD as categorical outcome variables with HR-ASD as reference category and the two CBCL 1.5–5 and C-TRF scales as predictor variables.

#### Relations Between ADOS-2 CS and the CBCL 1.5–5/C-TRF Scales (Aim 2)

Pearson’s *r* bivariate correlations between the ADOS-2 CS and the CBCL 1.5–5 and C-TRF for the HR group (HR-noASD and HR-ASD included) were conducted. Prior to the linear regression, assumptions of normality of residuals and homoscedasticity were inspected and found acceptably fulfilled. Multiple linear forced entry regressions were conducted with the CBCL 1.5–5 and C-TRF scores for the Withdrawn and PDP scales as predictors and the ADOS-2 CS as outcome variable.

## Results

### Categorical Comparisons (Aim 1)

For group differences, the separate ANOVAs in Table 3 show that preschool ratings on both the Withdrawn and PDP scales discriminated between the HR-ASD and the LR as well as the HR-noASD groups, with higher mean scores for the HR-ASD group. No difference was found between the HR-noASD and LR groups. For parents, a trend was found for the Withdrawn scale, indicating higher scores for the HR-ASD group than the LR group (*p* = .091) and non-significant results for other comparisons (*p*s .104–.557). For the parent PDP scale, the LR group had lower mean scores than the two other groups, however only as a trend for the HR-ASD vs. LR group (*p* = .059). There was no difference between the HR-ASD and HR-noASD groups.

**Table 3:** Group comparison on the Withdrawn and PDP subscales

	LR n = 14			HR-noASD n = 32			HR-ASD n = 10			Group comparisons		
	M	SD	Range	M	SD	Range	M	SD	Range	F* (df)	Corr *p*	Post hoc Games-Howell
C-TRFW	0.29	0.73	2	1.03	1.96	9	3.70	2.54	8	10.12 (2, 17)	.002	HR-ASD > (LR**; HR-noASD*)
CBCLW	0.57	0.85	2	1.47	2.06	8	2.30	2.21	7	2.79 (2, 21)	.084^†^	*ns*
C-TRFPDP	0.86	1.23	4	1.78	2.42	10	6.20	4.10	11	10.86 (2, 14)	.002	HR-ASD > (LR**; HR-noASD*)
CBCLPDP	0.86	0.95	3	2.59	3.19	15	6.20	6.27	20	4.55 (2, 12)	.045	LR < (HR-ASD^†^; HRnoASD*)

*LR* Low-risk siblings, *HR-noASD* High-risk sibling with no ASD, *HR-ASD* High-risk sibling with ASD, *C-TRFW* Caregiver-Teacher Report Form Withdrawn scale, *C-TRFPDP* Caregiver-Teacher Report Form Pervasive Developmental Problems, *CBCLW* Child Behavior Checklist Withdrawn scale, *CBCLPDP* Child Behavior Checklist Pervasive Developmental Problems, *Corr* corrected, F* Brown Forsythe’s *F*

^†^*p* ≤ .1; *p ≤ .05; **p ≤ .01

In the analyses to follow, we analysed how well the Withdrawn and PDP scales predicted group category by conducting two logistic regression analyses, one for each scale. Ratings by parents and preschool staff were entered in the model as predictors. As can be seen in Table 4, a substantial part of the variance in diagnostic status was explained by the CBCL 1.5–5 and TRF ratings (R^2^ range .28–.35). Further, the preschool ratings contributed uniquely to discriminating between the two HR groups for both scales tested (Withdrawn and PDP-scales). For the parent CBCL 1.5–5 result, the PDP scale differentiated between the HR-ASD and the LR groups but not the HR-ASD and the HR-noASD groups (*p* = .330). The parent Withdrawn scale made no significant contribution to the model (*p*s = .117–.656).

**Table 4:** Multinominal logistic regression

C-TRF/CBCL withdrawn^a^		95% CI for odds ratio		
Group	*b* (SE)	Lower	Odds ratio	Upper
LR vs. HR-ASD				
Intercept	2.06 (0.72)**			
C-TRF W	− 0.98 (0.46)*	0.15	0.38	0.94
CBCL W	− 0.45 (0.34)	0.33	0.64	1.24
HR-noASD vs. HR-ASD				
Intercept	2.26 (0.65)**			
C-TRF W	− 0.44 (0.17)**	0.46	0.65	0.91
CBCL W	− 0.09 (0.19)	0.62	0.91	1.33

C-TRF/CBCL pervasive developmental problems^b^		95% CI for odds ratio		
Group	*b* (SE)	Lower	Odds ratio	Upper
LR vs. HR-ASD				
Intercept	2.92 (0.87)*			
C-TRF PDP	− 0.55 (0.24)*	0.36	0.58	0.93
CBCL PDP	− 0.57 (0.29)*	0.32	0.56	1.00
HR-noASD vs. HR-ASD				
Intercept	2.83 (0.76)**			
C-TRF PDP	− 0.35 (0.13)**	0.54	0.70	0.91
CBCL PDP	− 0.09 (0.10)	0.75	0.91	1.11

LR n = 14; HR-noASD n = 32; HR-ASD n = 10

*LR *Low-risk siblings, *HR-noASD *High-risk sibling with no ASD, *HR-ASD* High-risk sibling with ASD, *C-TRFW* Caregiver-Teacher Report Form Withdrawn scale, *C-TRFPDP* Caregiver-Teacher Report Form Pervasive Developmental Problems, *CBCLW* Child Behavior Checklist Withdrawn scale, *CBCLPDP* Child Behavior Checklist Pervasive Developmental Problems, *CI* Confidence Intervals, *p ≤ .05; **p ≤ .01

^a^R^2^ = .28 (Cox & Snell), .32 (Nagelkerke). Model X^2^ (4) = 18.24, *p* = .001

^b^R^2^ = .35 (Cox & Snell), .41 (Nagelkerke). Model X^2^ (4) = 24.28, *p* < .001

### Continuous Relations in the HR Sample (Aim 2)

Table 5 presents zero-order correlations between the ADOS-2 CS and the CBCL 1.5–5/C-TRF scales for the HR group. The ADOS-2 CS were positively correlated with the preschool PDP ratings. The Withdrawn ratings showed a trend in the same direction as the PDP ratings, but did not reach statistical significance. The two parent CBCL 1.5–5 scales showed no significant correlations with the ADOS-2 CS.

**Table 5:** Correlations (Pearsons’s r) between ADOS-2 CS and the CBCL—C-TRF scores

	C-TRF preschool W	C-TRF preschool PDP	CBCL parent W	CBCL parent PDP
ADOS-2 CS *r*	.349^†^	.489***	.141	.216
CI 95%	.027–.680	.154–.754	− .148 to .481	− .094 to .493

High risk sample n = 42

*ADOS-2 CS* Autism Observation Schedule Comparison Score, *C-TRF* Caregiver-Teacher Report Form, *CBCL* Child Behavior Checklist, *W* Withdrawn scale, *PDP* Pervasive Developmental Problems scale

^†^*p* ≤ .1; ***p ≤ .001

Next, we conducted two linear regressions with CBCL 1.5–5 /C-TRF data from the two informants as predictor variables and the ADOS-2 CS as outcome variable. We did this separately for the PDP and the Withdrawn scales. Together, preschool C-TRF and parent CBCL 1.5–5 Withdrawn scores explained variance in the ADOS-2 CS, but only at trend level (R^2^ = .13, Adj R^2^ = .08, *F*(2,39) = 2.80, *p* = .073), no scale showing a significant unique contribution to the model (preschool Withdrawn: *p* = .193, 95% CI − .048–.935; parent Withdrawn: *p* = .711, 95% CI −.266–.576). In contrast, the model with the predictor variables preschool C-TRF PDP and parent CBCL 1.5–5 PDP was statistically significant (R^2^ = .24, Adj R^2^ = .20, *F*(2,39) = 6.14, *p* = .005) and the C-TRF PDP scale made a significant unique contribution to the model (β = 0.48, *p* = .031) explaining 19.3% of the variance in ADOS-2 CS whereas the CBCL 1.5–5 PDP scores made no significant contribution (β = 0.01, *p* = .94).

## Discussion

In this study, we investigated how ratings on the parent CBCL 1.5–5 and C-TRF Withdrawn and PDP scales are associated with autism symptoms by comparing high-risk children with and without diagnosis as well as low-risk children with typical development. We also investigated the relation between the CBCL 1.5–5 and C-TRF scales and severity measures of autistic symptoms (ADOS-2 CS) in the whole HR sample. Our results suggest that compared to parents, information from preschool staff more accurately discriminate between high-risk groups with and without autism and controls, and more accurately track autism symptoms in very young children.

Our results are in line with studies on the SRS in older children which found that teachers rated autism symptoms more accurately than parents, both regarding group differences and correlations with ADOS-2 CS (Constantino 2002; Constantino and Gruber 2012; Aldridge et al. 2012; Reszka et al. 2014; Duvekot et al. 2015). Our study shows that this advantage also applies for children as young as 3 years. This advantage may not be surprising, as teachers and care staff in preschool meet such a large range of children (Reed and Osborne 2013), enabling comparing children’s behavior in a more normative way than parents. Also, many of the behaviors that define ASD are linked to social interactions with peers which may be more easily assessed in preschool. Thus, the preschool staff could report more accurately simply because they experience more of the phenomena in question.

The results for the C-TRF PDP scale were significant both for the categorical and dimensional analysis (aim 1 and aim 2). The correlations between the C-TRF Withdrawn scale and the ADOS CS, while in the same direction as for the PDP scale, did not reach statistical significance. This could suggest that the ASD specific items found in the PDP scale (mainly of restricted and repetitive behaviors), not the common items represented in both scales (Table 2), contribute stronger to the accuracy of the C-TRF ratings in relation to autism symptoms (ADOS-2 CS).

Contrary to preschool ratings, for parent ratings no correlations were significant for the CBCL1.5–5 Withdrawn and PDP scales in relation to autism symptoms. Moreover, whereas preschool informants could differentiate between the HR-ASD group and the two groups with no ASD (HR-noASD and LR groups) on the Withdrawn and PDP scales, parents could only differentiate between the HR (HR-ASD and HR-noASD groups) and the LR group for the PDP scale. A similar but weaker pattern was also found for the CBCL 1.5–5 Withdrawn scale. Taken together, this suggests that while parents are able to discriminate between groups that differ substantially in terms of symptoms, they are less able to detect (or report) more subtle differences between affected and unaffected HR siblings. Apart from having less opportunity to compare different children’s behavior than preschool staff (and perhaps also seeing less relevant behaviors, as noted above), an additional reason for the parent disadvantage in the current study might be that the high risk group is special in the sense that all families have at least one older child with ASD taking part in a sibling study. It is possible that this experience biases the parents’ ratings of the younger child in ways that do not contribute to accurate reporting of symptoms. The older children may be very heterogeneous in their behavior which leads to a reference bias in the rating of the younger sibling. We are not aware of any study that has compared the accuracy of parent report for parents with and without older children with ASD. It should be noted however, that in the study by Rescorla et al. (2017) on 24-months-old HR siblings (of similar sample size) it was found that parents with an older child with ASD were indeed able to discriminate between the ASD group and the groups with no ASD (HR with no ASD and LR) for both the Withdrawn and PDP scales. The difference in results between our study and the study by Rescorla et al. may be due to cultural differences or age differences, the latter possibly reflecting the emergence of more symptomatology in the high-risk group from 2 to 3 years (Landa et al. 2013; Ozonoff et al. 2015), making differentiation at 3 years harder for parents in the HR group than at 2 years.

### Limitations and Future Directions

One limitation of this study is the small sample size. Yet, we would like to emphasize that this cannot explain why we found that the preschool informants were significantly better than the parents, i.e., showing the expected direction of differences. Also, as previously noted the high risk group is special in the sense that all families have at least one older child with ASD and are taking part in a sibling project. It is possible that this experience may bias the parents’ ratings of the younger child. Another limitation is that the former 13-item PDP scale (Achenbach and Rescorla 2000) was used in this study, comprising the item “afraid to try new things”. This particular item has been removed in the current renamed DSM-ASD scale (Achenbach 2014). However, as the rest of the 12 items are identical, a change in the main results due to the inclusion of this particular item would be unlikely.

Further studies are needed to evaluate how the current results generalize to other countries and contexts (Rescorla et al. 2012). In this study, the preschool staff comprised preschool teachers with a bachelor degree and care staff with upper secondary level qualifications. Many countries within the European Union have equivalent or even higher qualifications than in Sweden and this suggest that evaluation can be done equally well in these countries and that our results can be generalized to these countries. However, the results may not generalize to other countries that do not have staff with equivalent education. Moreover, in Sweden 93% of the 3-year-olds attend preschool and this could possibly have an impact on the current results. Nevertheless, many countries within the OECD have high attendance to preschool for children 3 to 4 years old (mean 80%) and 82% of all 3-year-olds within the European Union attend preschool which makes comparisons reliable (http://ec.europa.eu/eurostat). However, in the United States the attendance to pre-primary school for 3-year-olds is 42% which could make comparisons less reliable (https://nces.ed.gov). Of note though, the association between ratings on the PDP-scale and the ADOS for preschool staff in the current study is very similar to associations found on the SRS in research on teachers within the United States (Reszka et al. 2014; Azad et al. 2016; Schanding et al. 2012), speaking for compatible results.

Furthermore, it has been found that a subset of children in the HR-noASD group have deviant developmental behaviors and symptoms, while the rest have no symptoms despite being at familial risk (Messinger et al. 2013; Charman et al. 2017). Thus, in future studies to further assess the informants’ ability to discriminate between subgroups, groups could be split into four categories rather than the current three. Lastly, it would be of interest to investigate ratings by preschool staff on more specific autism measures such as the SRS-2 (Constantino and Gruber 2012) and the Repetitive Behavior Scale-Revised (Bodfish et al. 2000) in order to investigate if these comprehensive measures adds to the differentiation between groups in relation to the C-TRF PDP scale. This would also enable to study if both social and repetitive aspects are spotted at a clinical and subclinical level by preschool informants.

### Clinical Implications

Our result shows that compared to parents, preschool staff can better discriminate between groups, and they rate autistic behaviors more in accordance with expert observations (ADOS CS). Even if limitations with generalizing results need to be stressed, this points to the potential value of including information from preschool staff in early ASD assessment. Still, it is important to emphasize that ratings on C-TRF 1.5-5 may result in misclassification. For example, in our study, two out of 10 children with ASD had a score of zero on the preschool PDP scale, indicating limited sensitivity. More research is needed to clarify the reasons for preschool staff sometimes not identifying autism symptoms, whether it is related to the subtlety of symptoms or a matter of lack of knowledge and training or both (Långh et al. 2017; Engstrand and Roll-Pettersson 2014).

## Conclusion

We find that compared to parent ratings, ratings by preschool staff more accurately differentiate children with ASD from LR children and HR children without a diagnosis. Moreover, preschool staff rate ASD symptoms more in line with clinical observation. Parents can differentiate those who clearly have no autistic symptoms from those who have, but report less fine-grained distinctions between groups. Our results suggest that preschool staff are good at spotting clinical autism behavior in young children, and may contribute in important ways to the evaluation of ASD.
